# CUMULATIVE DIS/ADVANTAGE, COHORTS, AND ECONOMIC SHOCKS BEFORE AND AFTER: LIFE COURSE INEQUALITY AND THE RECESSION

**DOI:** 10.1093/geroni/igz038.1561

**Published:** 2019-11-08

**Authors:** Stephen Crystal, Naomi Zewde

**Affiliations:** 1 Rutgers University, New Brunswick, New Jersey, United States; 2 School of Social Work, Columbia University, New York, New York, United States

## Abstract

We examined age/cohort profiles of income inequality in the Survey of Consumer Finances for 2007, 2010, and 2016. Overall, the Gini coefficient decreased somewhat following the 2008 onset of the Recession, but rebounded by 2016 to a higher level than the 2007 baseline. Within-cohort inequality trends from 2007-2016 differed considerably across cohorts. For late baby boomers and millennials, inequality increased sharply – from .56 to .67 for the 1963-1972 birth cohort and from .49 to .60 for the 1973-1982 birth cohort. Inequality was higher at baseline for early baby boomers than later cohorts but did not increase as they moved into midlife and early late life. The Great Recession, with its large drops in asset values, initially led to slight reductions in inequality, but this effect was temporary. Late boomers and millennials experienced sharp increases in inequality following the Recession, presaging a very high-inequality late-life experience for these cohorts.

